# Genome-wide and molecular characterization of the *DNA replication helicase 2* (*DNA2*) gene family in rice under drought and salt stress

**DOI:** 10.3389/fgene.2022.1039548

**Published:** 2022-11-22

**Authors:** Bilal Saleem, Umer Farooq, Obaid Ur Rehman, Muhammad Aqeel, Muhammad Shahbaz Farooq, Muhammad Kashif Naeem, Safeena Inam, Wajya Ajmal, Amna Abdul Rahim, Ming Chen, Rabia Kalsoom, Muhammad Uzair, Sajid Fiaz, Kotb Attia, Hayat Ali Alafari, Muhammad Ramzan Khan, Guoping Yu

**Affiliations:** ^1^ National Institute for Genomics and Advanced Biotechnology, National Agricultural Research Centre, Islamabad, Pakistan; ^2^ Department of Bioinformatics, College of Life Sciences, Zhejiang University, Hangzhou, China; ^3^ School of Biological Sciences and Technology, Beijing Forestry University, Beijing, China; ^4^ National Key Facility for Crop Gene Resources and Genetic Improvement, Institute of Crop Sciences, Chinese Academy of Agricultural Sciences, Beijing, China; ^5^ Department of Plant Breeding and Genetics, The University of Haripur, Haripur, Pakistan; ^6^ Department of Biochemistry, College of Science, King Saud University, Riyadh, Saudi Arabia; ^7^ Department of Biology, College of Science, Princess Nourah Bint Abdulrahman University, Riyadh, Saudi Arabia; ^8^ National Nanfan Research Institute, Chinese Academy of Agricultural Sciences, Sanya, China; ^9^ China National Rice Research Institute, Hangzhou, China; ^10^ Hainan Yazhou Bay Seed Lab, Sanya, China

**Keywords:** rice, abiotic stress, DNA2, DNA damage-repair, gene expression

## Abstract

Rice plants experience various biotic (such as insect and pest attack) and abiotic (such as drought, salt, heat, and cold *etc.*) stresses during the growing season, resulting in DNA damage and the subsequent losses in rice production. *DNA Replication Helicase*/*Nuclease2 (DNA2)* is known to be involved in DNA replication and repair. In animals and yeast *DNA2* are well characterized because it has the abilities of both helicase and nuclease, it plays a crucial role in DNA replication in the nucleus and mitochondrial genomes. However; they are not fully examined in plants due to less focused on plants damage repair. To fill this research gap, the current study focused on the genome-wide identification and characterization of *OsDNA2* genes, along with analyses of their transcriptional expression, duplication, and phylogeny in rice. Overall, 17 *OsDNA2* members were reported to be found on eight different chromosomes (2, 3, 4, 6, 7, 9, 10, and 11). Among these chromosomes (Chr), Chr4 contained a maximum of six *OsDNA2* genes. Based on phylogenetic analysis, the *OsDNA2* gene members were clustered into three different groups. Furthermore, the conserved domains, gene structures, and *cis*-regulatory elements were systematically investigated. Gene duplication analysis revealed that *OsDNA2_2* had an evolutionary relationship with *OsDNA2_14, OsDNA2_5* with *OsDNA2_6,* and *OsDNA2_1* with *OsDNA2_8.* Moreover, results showed that the conserved domain (AAA_11 superfamily) were present in the *OsDNA2* genes, which belongs to the DEAD-like helicase superfamily. In addition, to understand the post-transcriptional modification of *OsDNA2* genes, miRNAs were predicted, where 653 miRNAs were reported to target 17 *OsDNA2* genes. The results indicated that at the maximum, *OsDNA2_1* and *OsDNA2_4* were targeted by 74 miRNAs each, and *OsDNA2_9* was less targeted (20 miRNAs). The three-dimensional (3D) structures of 17 OsDNA2 proteins were also predicted. Expression of *OsDNA2* members was also carried out under drought and salt stresses, and conclusively their induction indicated the possible involvement of *OsDNA2* in DNA repair under stress when compared with the control. Further studies are recommended to confirm where this study will offer valuable basic data on the functioning of *DNA2* genes in rice and other crop plants.

## Introduction

Rice plants experience various biotic (such as insect and pest attack) and abiotic (such as drought, salt, heat, and cold *etc.*) stresses during the growing season, resulting in DNA damage and the subsequent losses in rice production. Homologous recombination is essential for replication, DNA repair pathways, and the exchange of genetic substances between parent chromosomes during meiosis (Li, 2008; Kuzminov, 2011). The complex reorganization of DNA structures is typically organized into several stages, and the success rate of completing these stages is entirely related to the activities of multiple helicase enzymes (Wu, 2012; Huselid and Bunting, 2020). Helicases of several families are organized to process the broken ends of DNA structures, and are also involved in the subsequent disassembly and formation of recombinant intermediate materials essential for the template-based repair of DNA structures (Raney et al., 2013; Croteau et al., 2014; Tisi et al., 2020). Therefore, the loss of recombinant-linked helicase functionality can result in genome disorder, higher risks of tumor forging, and subsequent cell death (Yousefzadeh et al., 2021). Certain helicases are associated with the anti-recombinase effects that influence the recombination efficiency, ultimately leading to other pathways directed toward repairing broken ends of DNA (Wu and Hickson, 2006; Li and Heyer, 2008). Several helicases are also responsible for adjusting the relative repair outputs for noncrossover and crossover. A typical increase in the utilization of recombination occurs during the collision of transcription material and replication forks, or when it comes across lesions in the DNA template (Nguyen et al., 2015). Amazingly, successful recombination in such situations is also regulated by helicases, permitting optimized cell growth while maintaining genome integrity (Saada et al., 2018).

During developmental processes, the unrelenting activities of apical meristems organize organ morphogenesis (Traas, 2018; Marconi and Wabnik, 2021). The development of apical meristems is maintained and promoted by cell division in meristematic areas (Murray et al., 2012; Geng et al., 2022). Plant meristems are accompanied by stem cells, and have a strong regenerative ability; they maintain plant growth mechanisms and produce new plant organs such as stems, leaves, flowers, and roots (Chang et al., 2020). The final forms of the organs and the overall plant architecture mainly depend on the temporal and spatial regulation of cell proliferation in meristems.

*DNA2*, typically called the DNA replication helicase/nuclease two protein, occurs in both the mitochondria and nucleus, where it performs the roles of helicase and ATPase-dependent nuclease (Zhou et al., 2015; Zheng et al., 2020). During the 1980s, the DNA2 protein was first reported in the yeast *Saccharomyces cerevisiae* (Budd et al., 2000)*.* Because it has the abilities of both helicase and nuclease, it plays a crucial role in DNA replication in the nucleus and mitochondrial genomes (Dumas et al., 1982; Budd and Campbell, 1997). In humans, it is known as *DNA replication ATP-dependent helicase/nuclease DNA2* (Masuda-Sasa et al., 2006). *DNA2* essentially shares an important purpose in the removal of long flaps during DNA replication and DNA LP-BER (long-patch base excision) repair. Moreover, it interacts with flap endonuclease 1 (FEN1) and replication protein A (RPA) (Kleppa et al., 2012). *DNA2* has the ability to promote the reactivation of the prehended replication fork along with BLM (Bloom syndrome protein) and WRN (Werner syndrome ATP-dependent helicase) (Machwe et al., 2006; Sturzenegger et al., 2014). *DNA2* assists in the removal of primers during strand displacement replication in the mitochondria (Zheng et al., 2008). Additionally, DNA2 is considered to act as a key to protein-sharing activities in complex DNA damage repair. Moreover, it is accompanied by a double stranded break (DSB) and a 50 reactive adduct resulting from a chemical group attached to DNA 50 ends, produced by ionization of the radiation (Pawłowska et al., 2017). In human and animal cells, the key role of *DNA2* in general cell cycle maintenance proposes its generalized function in genomic integrity. Therefore, for human and animal cells, it is essential for disease therapy.

During the repair of broken DNA ends, *DNA2* intercedes the 59-end resection of DNA by splitting the 59-single-stranded DNA, ssDNA, with the assistance of RPA and Sgs1. Subsequently, it acts as helicase, whose function is mediated by RPA, and can disentangle DNA along the production of an ssDNA substrate for DNA2 (Cejka et al., 2010; Ruff et al., 2016; Bonetti et al., 2018). Homologs of RPA and Sgs1 are also preserved in plants, with essential duties in different pathways, such as DNA repair (Chatterjee and Walker, 2017; Verma et al., 2020; Wang et al., 2021). Yeast DNA2 is occupied by the compound nuclear localization signal (NLS) sequences, Pat4 and Pat7, and is decentralized to the nucleus (Jia et al., 2016; Meng et al., 2019). Yeast DNA2 mutants are sensitive to DNA damage factors, including X-ray and UV irradiation, and methyl methane sulfonate (MMS) (Bae et al., 2001; Jia et al., 2016). Moreover, yeast DNA2 exhibits DNA repair activities by assisting homologous recombination. In mammalian cells, DNA2 participates in DNA repair and replication, whereas in humans, a reduction in hDNA2 delays cell division as well as the entire cell cycle (Duxin et al., 2009; Bailey et al., 2019; Hudson and Rass, 2021).

On a broader level, *DNA2* is thought to have essential roles in DNA repair and replication, along with maintaining nuclear genomic DNA and mitochondrial integrity in fungi and animals (Duxin et al., 2009; Gredilla et al., 2012). However, the role of *DNA2* in plants has not yet been investigated due to less focus on plants damage repair. Therefore, this study performed with objectives of genome-wide identification and characterization of *OsDNA2* genes in the rice genome along with their differential expression analysis.

## Materials and methods

### Evidencing the identification of *OsDNA2*

Phytozome database was used to obtain sequences of DNA2 proteins (Zhang et al., 2012). In the rice genome (*Oryza sativa* IRGSP-1.0), the Hidden Markov Model (HMM) profiles of DNA2 domain from the Pfam (protein family) database were used to scan the predicted proteins using HMMERv3 (Prince and Pickett, 2002). By using HMM model in HMMERv3, the protein sequences of rice DNA2 were aligned. For the confirmation of the presence of DNA2 conserved domain, the putative *DNA2* gene core sequences were verified by searching against the SMART (http://smart.embl-heidelberg.de/) and Pfam database (https://pfam.xfam.org/). Protein sequences of *Zea mays*, *Hordeum vulgare*, *Pennisetum glaucum*, and *Oryza sativa* were obtained from previous studies (Liu and Widmer, 2014; Guo et al., 2017), and TAIR (https://www.arabidopsis.org/) source was used to download information of Arabidopsis *DNA2* gene family protein sequences and annotation. ExPASy (https://www.expasy.org/) online server was used to obtain molecular weight, GRAVY, Iso-electric point information for *OsDNA2* (Gasteiger et al., 2003).

### Chromosomal location, gene structural, and phylogenetic analysis

Rice genomic database in phytozome was accessed to get the *DNA2* genes genomic coordinated on rice chromosomes. All *OsDNA2* were present on the eight chromosomes of rice genome. Protein sequences of DNA2 in Arabidopsis, rice, maize, barley, and millet were aligned using clustalW. Bootstrap 1000 replications were used to generate phylogenetic tree using maximum likelihood (ML) method in MEGA 10. Coding sequence was compared with the corresponding full-length sequence for the identification of intron insertion sites in the *DNA2* genes. This identification was performed by using Gene Structure Display Server (Guo et al., 2007). The analysis of conserved *DNA2* motifs was performed by using MEME Suit (Multiple EM for Motif Elicitation) Version 4.12.0 (http://meme-suite.org/tools/meme) (Rehman et al., 2022). Following parameters were used for this analysis: ten motifs to be found with motif width between 10 and 200; site distribution was one occurrence per sequence or set at zero (at most one occurrence for each motif was allowed for each sequence), whereas the maximum number of motifs was set to 10 (Bailey et al., 2009). TBtools (https://bio.tools/tbtools) program was used for further analysis of MEME results (Chen et al., 2020). Unipro UGENE software package (Okonechnikov et al., 2012), helped to examine the conserved domains of OsDNA2 proteins. This aligned the sequences by the ClustalW algorithm and conserved regions were displayed in the form of color patterns which differentiated each amino acid based on physiochemical properties. OsDNA2 protein sequences in SMART database containing Pfam domain search options, was used to perform protein domain analysis, and confirmation was carried out through the InterPro database (Letunic et al., 2012; Blum et al., 2021).

### MicroRNA, gene ontology, *cis*-elements, collinearity and synteny prediction in *OsDNA2*

MicroRNAs (miRNAs) interacting with the *DNA2* genes were predicted form the available rice miRNA reference sequences by submitting genome sequences of *OsDNA2* to the psRNATARGET server (https://www.zhaolab.org/psRNATarget/). The visualization of miRNAs and *OsDNA2* gene was done with Cytoscape software (https://cytoscape.org/). Online tool gProfiler (https://biit.cs.ut.ee/gprofiler/gost, accessed on 20 July 2022) was used to conduct gene ontology (GO) analysis of OsDNA2 protein sequences with default parameters. The rice genome database was downloaded from RGAP (https://cottonfgd.org/) to get the *DNA2* promoter region sequence containing 2000 bp upstream of the inhibition codon (ATG). The prediction of regulatory elements in the *DNA2* promoter regions was carried out by using online tool PlantCARE (http://bioinformatics.psb.ugent.be/webtools/plantcare/html/) and visualized by TBtools. For the sequence similarity patterns evaluation, synteny analysis and sequence identity visualization were performed using the TBtools (Rahim et al., 2022). For gene duplication, homologous gene pair were calculated with the help of Ka/Ks calculator 2.0. For Synteny analysis, genome sequence (FASTA) and annotation files (gff/gtf) were used in One-step MCScanX toolkit in the TBtools.

### Subcellular localization and 3D protein structure prediction of OsDNA2

CELLO v.2.5: subCELlular LOcalization predictor was used to predict the sub-cellular location of DNA2 family (Yu et al., 2014). Protein sequences were used as input and output results were further analyzed/visualized by using TBtools software. Amino acid sequences of OsDNA2 proteins were used for the prediction of 3D structures by utilizing the SWISS-MODEL database (https://www.swissmodel.expasy.org) (Waterhouse et al., 2018), while visualization of such predicted structures was carried out with the help of Pymol software (https://pymol.org/2/). Ramachandran Plot—Zlab, (https://zlab.umassmed.edu/bu/rama/), was applied for the confirmation of predicted 3D models of OsDNA2 proteins (Anderson et al., 2005).

### Expression analysis of *OsDNA2* under abiotic stress

RNA seq data for drought and salt stresses were assessed from online data bases which are publically available with Bio-projects GSE145869 and GSE167342 (Tarun et al., 2020; Bundó et al., 2022). In these studies, Nil-95 and Swarna genotypes were used as drought tolerant and sensitive, while IL22 and PL12 were used as a salt tolerant and sensitive rice genotype, respectively. FPKM (fragments per kilobase of transcript per million mapped reads) values were extracted and heatmaps were generated in TBtools. Furthermore, the rice genotype IR-6 was used in this study under drought and salt stresses. Plants were grown under normal conditions for 2 weeks. Then drought and salt stresses were applied to two batches of plants and one batch was kept as a control. Three biological replicates for each treatment were used. Gene specific primers of selected *OsDNA2* genes along with drought and salt reported genes (*OsEm1* and *bZIP23*) for qRT-PCR are presented in Supplementary Table S1. For this purpose, 1 g leaf tissues from control and treated samples were grinded in liquid nitrogen and used for RNA extraction. Total RNA was extracted with the help of TRIzol method. Complementary DNA (cDNA) was also synthesized from the 800 ng extracted RNA with the help of reverse transcriptase-III, first strand cDNA Synthesis Kit (K1691, Thermo Scientific Revert Aid). StepOne RT-PCR (Applied Biosystems^®^ 7900 H T Fast RT-PCR) was used for quantification. *OsActin* was used as a reference gene and ^2−ΔΔCT^ method was used for expression calculations (Uzair et al., 2021).

## Results

### Delineation of *DNA2* gene family in rice

**FIGURE 1:**
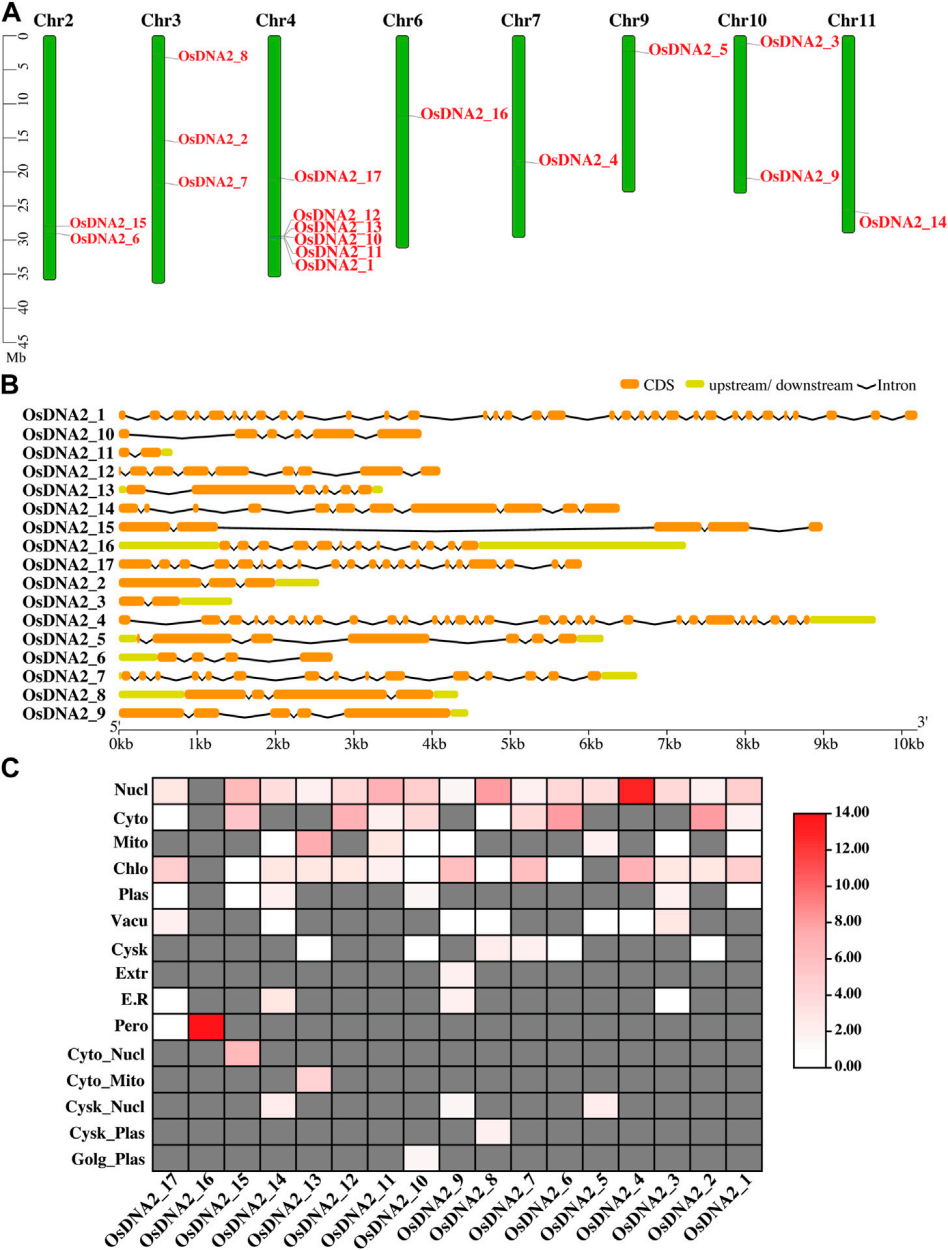
Prediction of *DNA2* gene members in rice genome. **(A)** Distribution of *OsDNA2* members on respective chromosome. Chr represent the chromosome. Left side of the figure scale was used in mega base (Mb). **(B)** Gene structure of *OsDNA2* members. Orange color shows the CDS, light green shows upstream/downstream region, and black line represent the introns. **(C)** *In-silico* prediction of subcellular location of OsDNA2. Proteins were shown on the right side of the figure. Nucl = nucleus, Cyto = cytoplasm, Mito = mitochondria, Chlo = chloroplast, Plas = plasma-membrane, Vacu = vacuole, Cysk = cytoskeleton, Extr = extracellular, E.R = endoplasmic reticulum, Pero = peroxisome, and Golg = Golgi apparatus.

Wheat DNA2 protein (TraesCS2A02g301600) and Arabidopsis (AT2G03270) sequences were used as queries to identify *DNA2* genes in the rice genome. From these analysis, 17 *OsDNA2* members were found (Figure 1A, Supplementary Table S2). *OsDNA2* was distributed on the basis of its physical position on eight chromosomes (2, 3, 4, 6, 7, 9, 10, and 11). Of these chromosomes, Chr4 had the maximum number of six *OsDNA2* genes, followed by Chr3, which had three genes. Chr2 and Chr10 each had two members, whereas the rest of the chromosomes had a single member. Chr9 was the shortest one which had *OsDNA2_5*. This structure plays an important role in the expression of genes. For this purpose, we also checked the gene structures and found that the number of introns varied from 1 to 32 (Figure 1B). The maximum number of introns was found in *OsDNA2_1*, whereas the minimum was found in *OsDNA2_11*. The CDS length extended from 399 bp (*OsDNA2_11*) to 3624 bp (*OsDNA2_1*). Similarly, the protein length, protein molecular weight, and number of exons varied from 133 aa (*OsDNA2_11*) to 1208 aa (*OsDNA2_1*), 132.72854 KDa (*OsDNA2_1*) to 14.19668 KDa (*OsDNA2_11*), and 2 (*OsDNA2_11* and *OsDNA2_3*) to 33 (*OsDNA2_1*), respectively (Table 1). A total of five genes (*OsDNA2_1, OsDNA2_2, OsDNA2_4, OsDNA2_7,* and *OsDNA2_17*) out of 17 were found on the positive strand. The isoelectric points varied between 4.2510 (*OsDNA2_11*) and 11.6011 (*OsDNA2_3*). The charge on a protein molecule depends on the ionizable groups and their pKa values. The protein becomes negatively charged when the pH becomes higher than the pI. In the present study, only four *OsDNA2* genes (*OsDNA2_2, OsDNA2_8, OsDNA2_10,* and *OsDNA2_11*) were negatively charged (Table 1). All members of the *OsDNA2* gene family, except *OsDNA2_7*, showed negative GRAVY, indicating that they are hydrophilic in nature (Table 1). Additionally, the subcellular localization of all OsDNA2 proteins was determined. The results showed that most of the genes were located in the nucleus, cytoplasm, mitochondria, and chloroplasts (Figure 1C). Meanwhile, the subcellular localization of *OsDNA2_16* was predicted in the peroxisomes (Figure 1C).

**TABLE 1:** Physico-chemical properties of *OsDNA2* gene members.

Transcript id	Gene name	Chr	Start	End	Str	CDS (bp)	Protein length (A.A)	Number of exons	Protein MW. (KDa)	pI	Charge	GRAVY
Os04g0588200	*OsDNA2_1*	04	29719544	29729743	+	3624	1208	33	132.72854	7.0511	12.5	-0.231
Os03g0387000	*OsDNA2_2*	03	15417019	15419577	+	1803	601	03	69.42784	5.1475	-13.5	-0.545
Os10g0118900	*OsDNA2_3*	10	1193347	1194794	-	459	153	02	17.09237	11.6011	9.5	-0.591
Os07g0495900	*OsDNA2_4*	07	18555542	18565210	+	3366	1122	29	123.49103	7.8445	23.0	-0.432
Os09g0130800	*OsDNA2_5*	09	2354862	2361051	-	2946	982	07	108.61926	8.3372	21.5	-0.329
Os02g0704300	*OsDNA2_6*	02	29059400	29062130	-	996	332	04	35.65298	7.1962	4.5	0.117
Os03g0586900	*OsARR-B7*	03	21673719	21680339	+	1956	652	15	71.30491	8.1619	9.0	-0.296
Os03g0160400	*OsDNA2_8*	03	3215854	3220186	-	2880	960	05	108.61155	5.0487	-22.5	-0.449
Os10g0537600	*OsDNA2_9*	10	20940762	20945224	-	2958	986	05	110.67024	6.5277	0.5	-0.670
Os04g0582700	*OsDNA2_10*	04	29438046	29441912	-	1752	584	06	66.15339	5.8404	-5.0	-0.427
Os04g0582900	*OsDNA2_11*	04	29448667	29449352	-	399	133	02	14.19668	4.2510	-9.0	-0.023
Os04g0582000	*OsDNA2_12*	04	29412733	29416839	-	2400	800	09	89.21547	7.9661	13.0	-0.347
Os04g0582600	*OsDNA2_13*	04	29431012	29434383	-	2154	718	06	80.96854	9.2093	29.5	-0.177
Os11g0649000	*OsDNA2_14*	11	25840593	25846990	-	3561	1187	11	130.11185	7.2920	11.5	-0.238
Os02g0684150	*OsDNA2_15*	02	27970821	27979810	-	2502	834	05	93.07151	8.1764	15.5	-0.284
Os06g0310200	*OsDNA2_16*	06	11781838	11789081	-	1527	509	18	55.71116	7.0864	4.0	-0.230
Os04g0424200	*OsDNA2_17*	04	20966881	20972795	+	2769	923	20	102.73190	7.3698	8.5	-0.294

A.A, amino acid, Chr = Chromosome, CDS , coding sequence; GRAVY , grand average of hydropathicity index; KDa, kilo Daltons; MW, molecular weight, pI = Iso-electric Point, Str = Strand.

### Motifs and domain analysis, and phylogenetic association among *DNA2* genes

When characterizing newly identified proteins, it is very important to understand the motifs and domains of that specific protein. In the present study, motifs were predicted for all *OsDNA2* genes using MEME (Bailey et al., 2009). For this purpose, we used the protein sequence of OsDNA2 and found ten conserved motifs (Figure 2A). The lengths of the predicted motifs varied between 20 and 39 amino acids (Supplementary Figure S1). Motif five was present in all genes except *OsDNA2_2, OsDNA2_6,* and *OsDNA2_12.* Motifs 1, 2, 3, and six were conserved among all members. Similarly, we also examined the domains in *OsDNA2* members (Figure 2B). The conserved domain in the *OsDNA2* genes of rice is the AAA_11 superfamily (Pfam: PF13086), which belongs to the DEAD-like helicase superfamily involved in the unwinding of ATP-dependent RNA or DNA.

**FIGURE:**
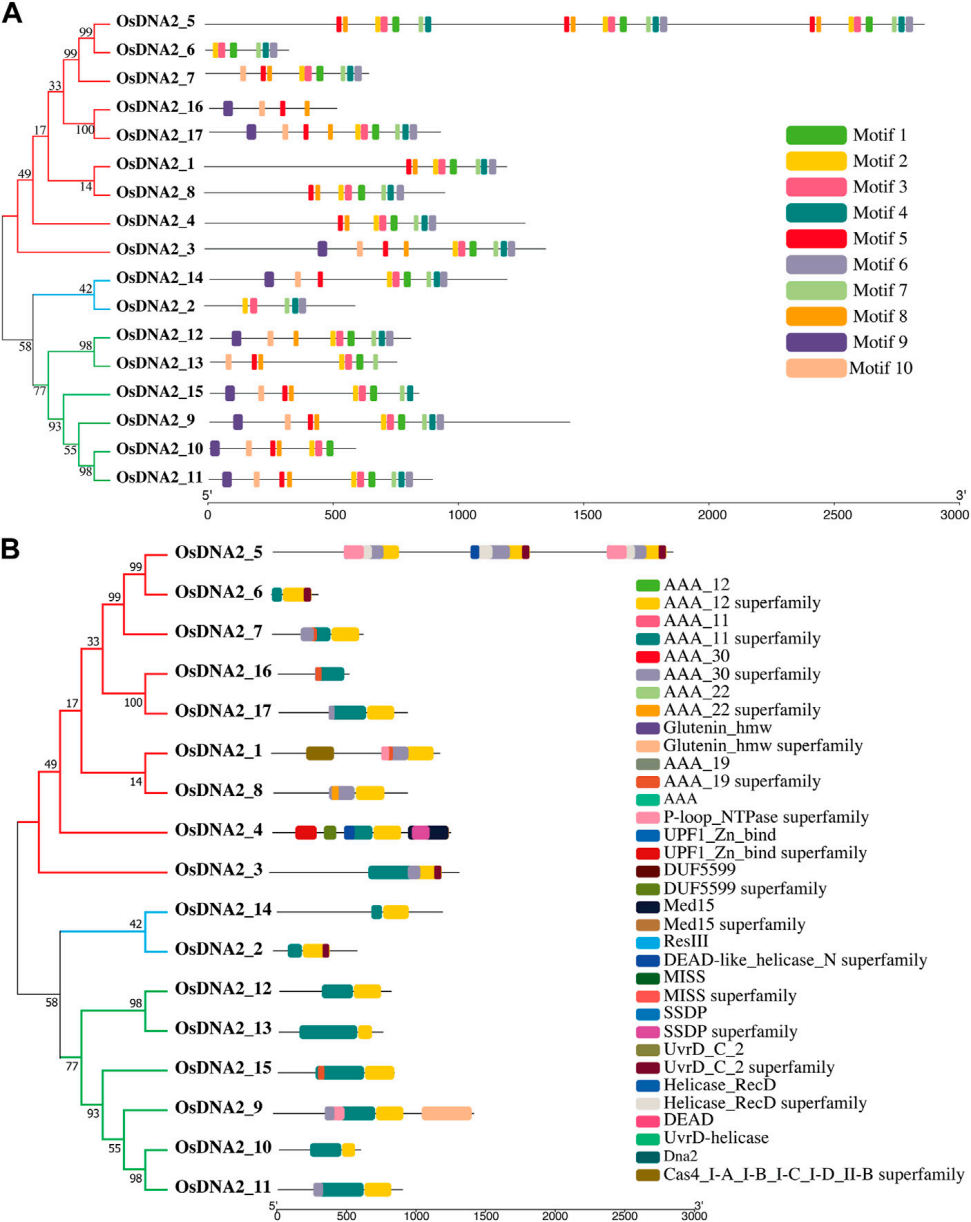
2OsDNA2 members were divided into three groups. Prediction of motifs **(A)** and domains **(B)** in OsDNA2 members. Each motif and domain were represented by different colors.

**FIGURE 3:**
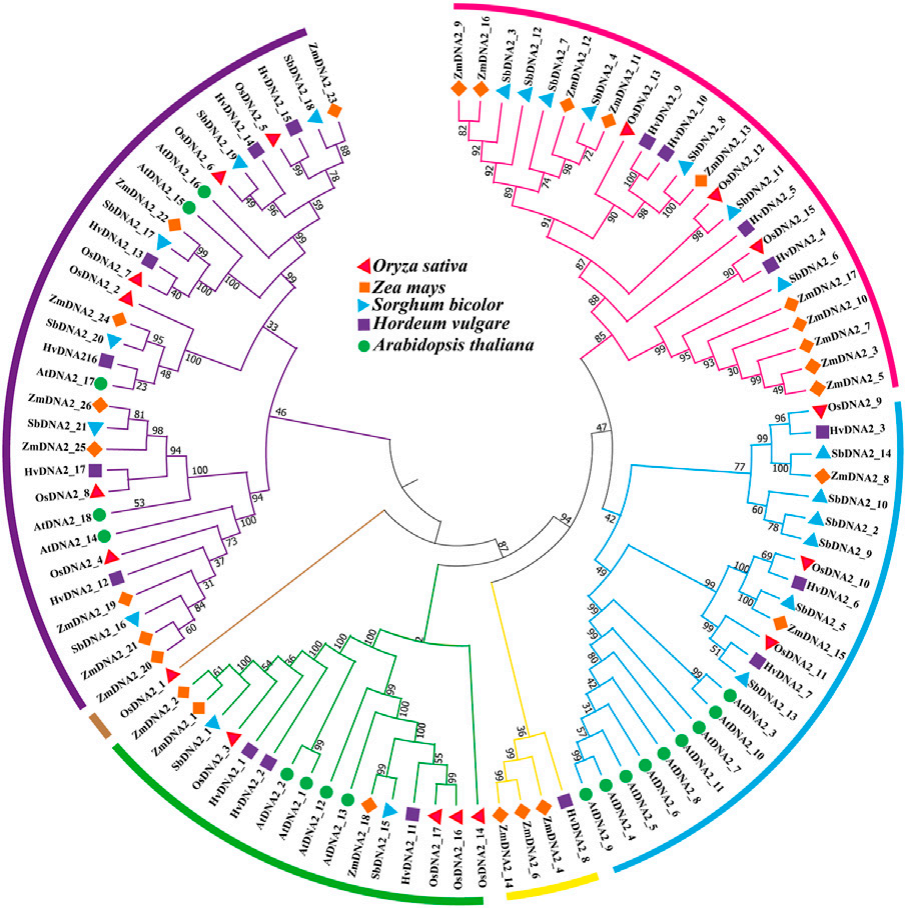
Evolutionary relationship among DNA2 members of Oryza sativa, Zea mays, Hordeum vulgare, and Arabidopsis thaliana. For the construction of phylogenetic tree, a maximum likelihood (ML) method was used with 1000 bootstrap values.

In the current study, the evolutionary associations among *OsDNA2s, ZmDNA2s, SbDNA2s, HvDNA2s*, and *AtDNA2s* were assessed (Figure 3). The results revealed that 99 *DNA2* molecules were clustered into six main clusters (C1 = pink, C2 = blue, C3 = yellow, C4 = green, C5 = brown, and C6 = purple). Cluster six contained a maximum of six *OsDNA2* genes (*OsDNA2_2* and *OsDNA2_4–8*). Interestingly, Cluster five contained only one, *OsDNA2_1*. Overall, OsDNA2 showed a closer association with ZmDNA2, HvDNA2, and SbDNA2 than with AtDNA2 in each cluster.

### Investigating synteny and collinearity among *DNA2* genes

**FIGURE 4:**
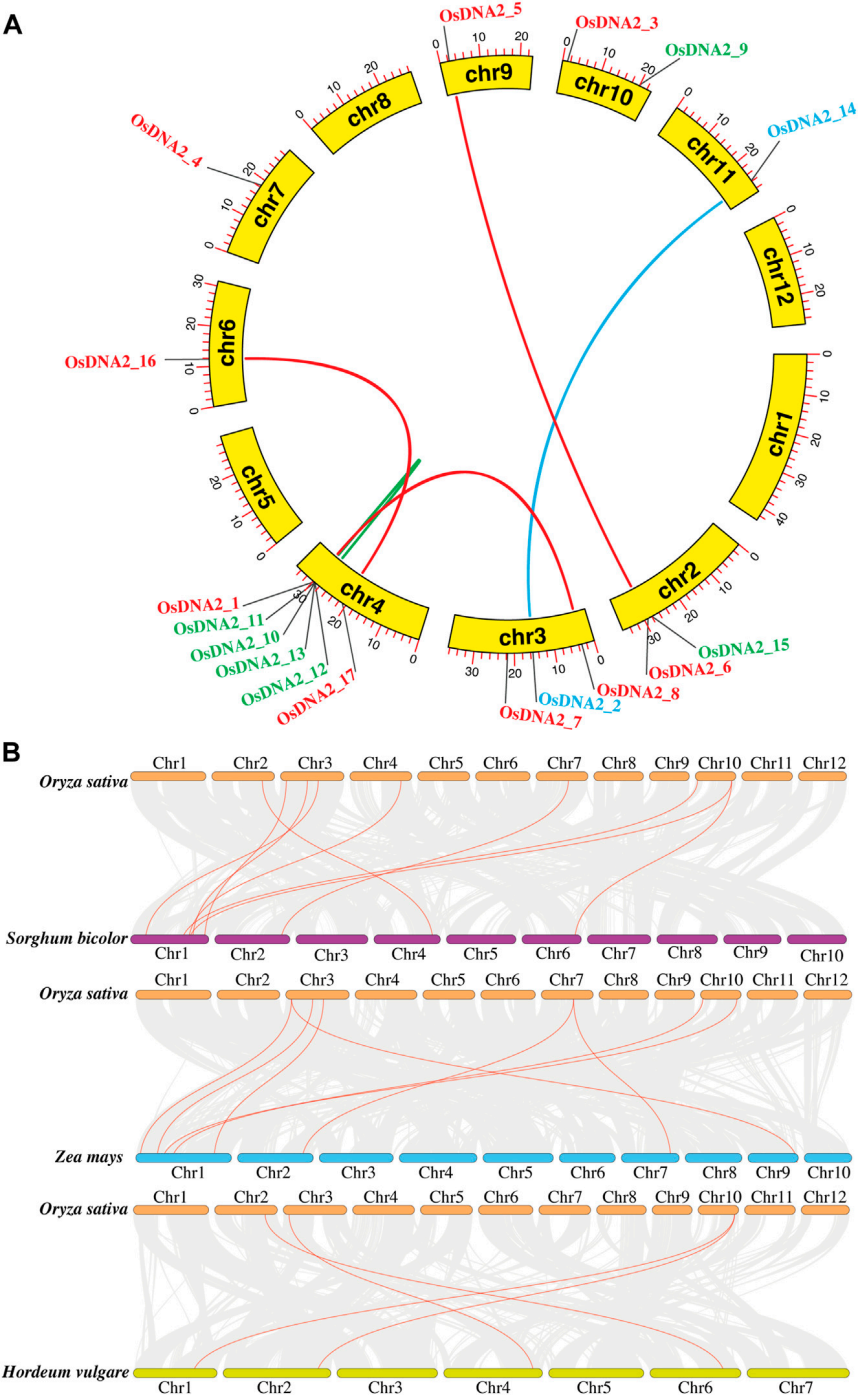
Identification of OsDNA2 gene duplication. **(A)** Synteny analysis and **(B)** Collinearity analysis of DNA2 genes amongst Oryza sativa, Sorghum bicolor, Zea mays, and Hordeum vulgare.

To estimate the evolutionary relationship among DNA2 members of *Oryza sativa*, a synteny analysis of DNA2 protein sequences was conducted (Figure 4A and Supplementary Table S2). These analyses were performed to study gene duplication using TBtools for the 17 predicted *OsDNA2* (Chen et al., 2020)*.* For gene duplication, Ka/Ks values were calculated, and the results revealed that six gene pairs, including *OsDNA2_1*-*OsDNA2_8*, *OsDNA2_2*-*OsDNA2_14*, *OsDNA2_5*-*OsDNA2_6*, *OsDNA2_10*-*OsDNA2_11*, *OsDNA2_12*-*OsDNA2_13*, and *OsDNA_16*-*OsDNA2_17* could be duplicated in the rice genome (Supplementary Table S3). Similarly, we also performed collinearity analysis among *Oryza sativa*, *Sorghum bicolor*, *Zea mays*, and *Hordeum vulgare*. The results revealed that rice *DNA2* genes were more collinear with sorghum than with maize and barley, indicating that whole genome or segmental duplication was involved in *OsDNA2* gene family progression (Figure 4B).

### Promoter analysis of *OsDNA2* genes

**FIGURE 5:**
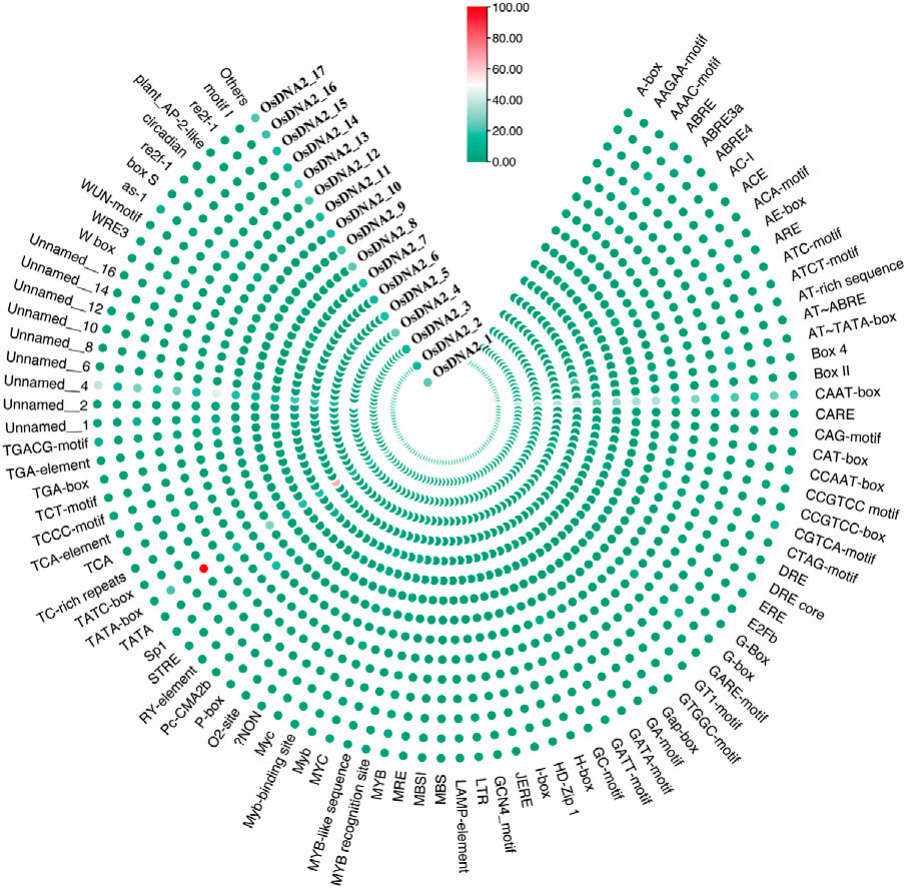
Prediction of Cis-Elements in 2 kb upstream region of OsDNA2 coding sequences. Scale bar was used to indicate the presence of numbers of specific elements in that particular DNA2 genes.

It was previously reported that the promoter region is the control center for the expression and regulation of genes (Rasool et al., 2021). Promoters are also known as *cis*-regulatory elements in DNA. The 2-kb upstream region of each *OsDNA2* gene was subjected to the PLACE database, and the results revealed that more than 85 different types of *cis*-acting elements and nine unnamed types of elements were detected (Figure 5, Supplementary Table S4). CAAT-box, TATA-box, and unnamed were the most identified elements for the *OsDNA2* genes. Furthermore, different stress-related regulatory elements, such as CGTCA-motif, G-box, Sp1, GATA-motif, I-box, GT1-motif, and AT-rich, were detected. Similarly, hormone-related *cis*-regulatory elements, such as TATC-box, ABRE, CGTCA-motif, P-box, and TGACG-motif, were also detected. ABRE was detected in all members of *OsDNA2*, except for *OsDNA2_6* (Figure 5). The GCN4-motif, GC-motif, O2-box, and ARE were detected in the different *OsDNA2* members, indicating that these genes are involved in cellular development. Notably, it has been reported that the GCN4-motif and O2-box are involved in the expression of endosperm and zein metabolism, respectively.

### Gene ontology (GO) and MicroRNA (miRNA) targeting *OsDNA2* genes

**FIGURE 6:**
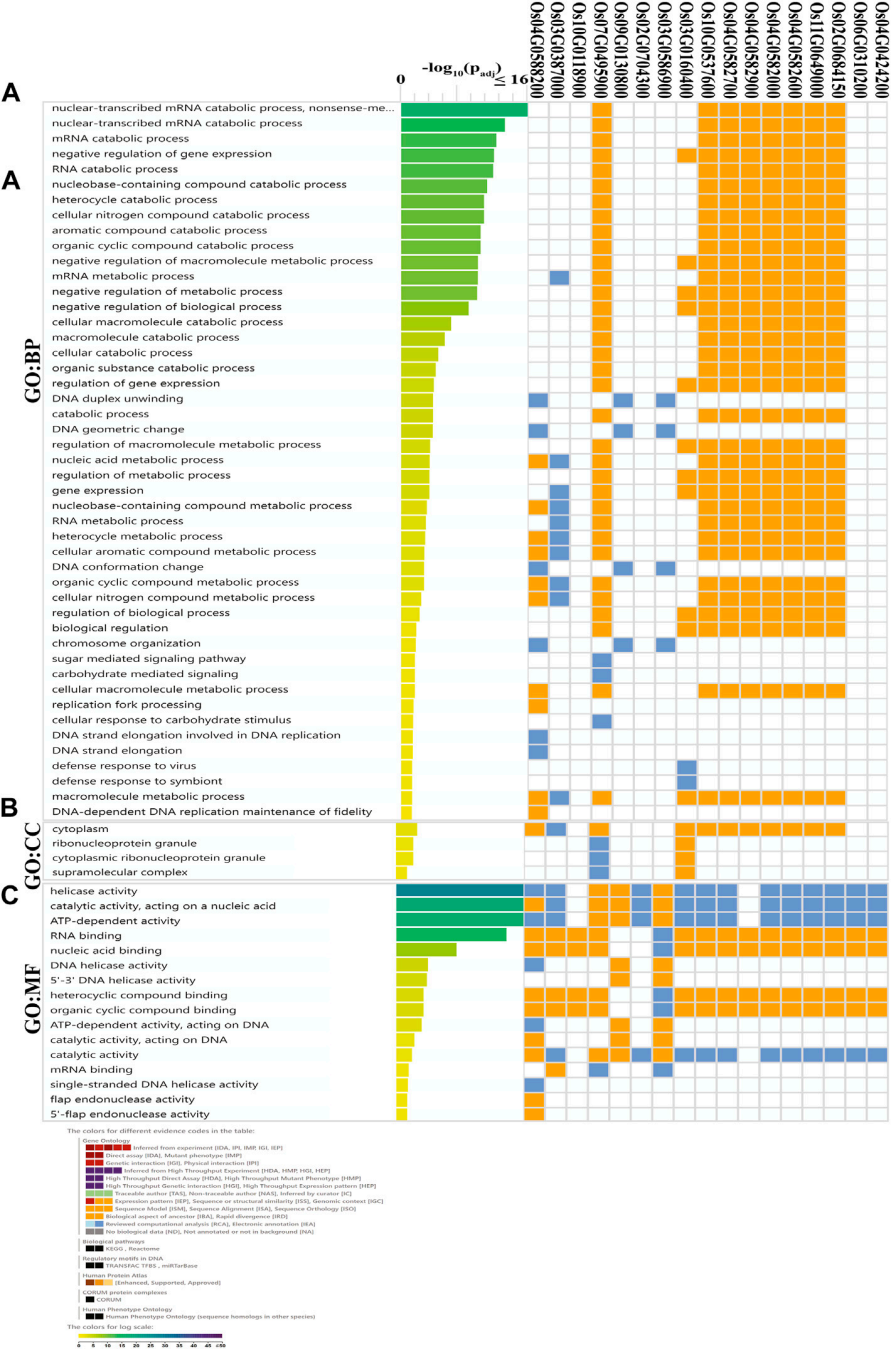
Gene ontology (GO) analysis of OsDNA2 genes was performed. OsDNA2 genes were involved in biological processes (BP, **(A)**, cellular components (CC, **(B)**, and molecular functions (MF, **(C)**.

GO annotation was used for the practical investigation of *OsDNA2* genes. *In silico* characterization based on functions was conducted, which revealed three types of biological processes (BPs), cellular components (CCs), and molecular functions (MFs) (Figure 6, Supplementary Table S5). Further analysis of the BPs’ annotations revealed that most of the terms were related to DNA replication. Similarly, the MFs also showed DNA helicase, and catalytic and ATP-dependent activities. Based on these findings, we concluded that *OsDNA2* genes play an essential role in DNA replication.

**FIGURE 7:**
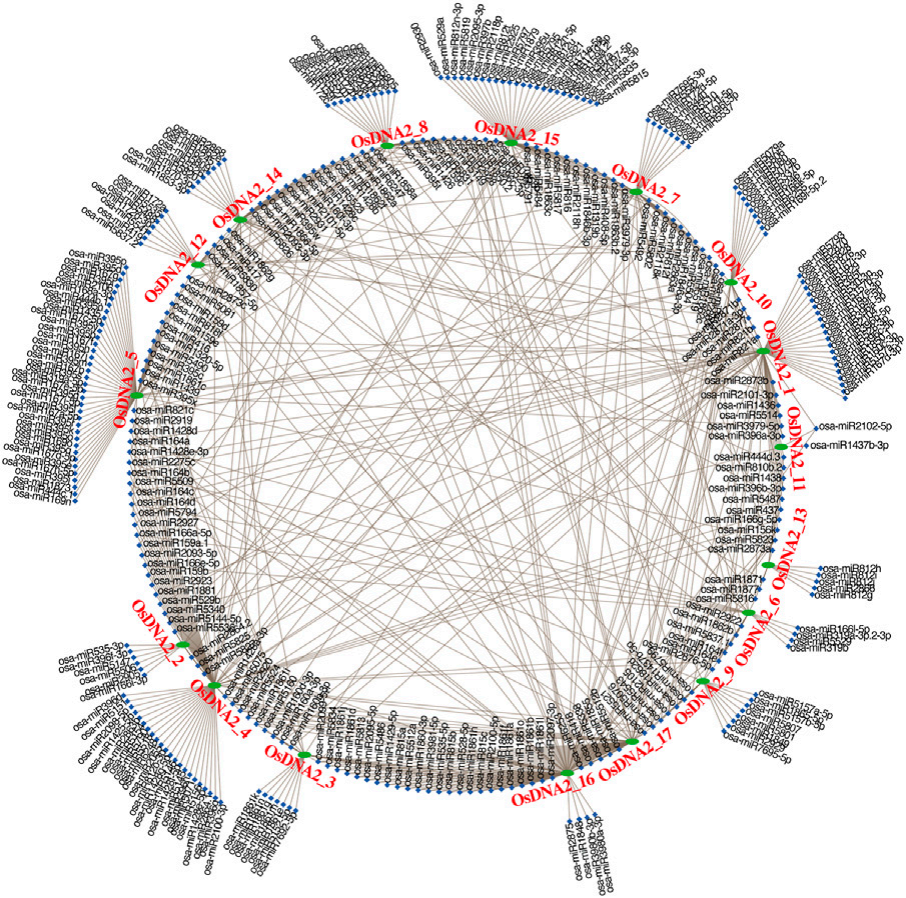
7Prediction of MicroRNAs (miRNAs) shows how they target the respective OsDNA2 genes. Green oval shapes represent the OsDNA2 genes, blue rectangles represent the miRNAs, and brown lines represent the interaction among the miRNAs and OsDNA2 genes.

Over the past few decades, many studies have shown that miRNAs play essential roles in the regulation of genes in specific environments. Thus, to understand the post-transcriptional modification of *OsDNA2* genes, we identified 653 miRNAs targeting 17 *OsDNA2* genes (Figure 7, Supplementary Table S6). The results indicated that at the maximum, *OsDNA2_1* and *OsDNA2_4* were targeted by 74 miRNAs each, and *OsDNA2_9* was less targeted (20 miRNAs). Nineteen members of the osa-miR164 family targeted four genes (*OsDNA2_1, OsDNA2_4, OsDNA2_6*, and *OsDNA2_14*). Furthermore, the single miRNAs, such as osa-miR6255, osa-miR6245, osa-miR6246, and osa-miR6248, targeted *OsDNA2_10, OsDNA2_14, OsDNA2_10*, and *OsDNA2_4*, respectively. Future studies will be required to validate the functions of these miRNAs and their target genes to understand their biological interactions in the rice genome.

### 3D-protein structural analysis of OsDNA2

**FIGURE 8:**
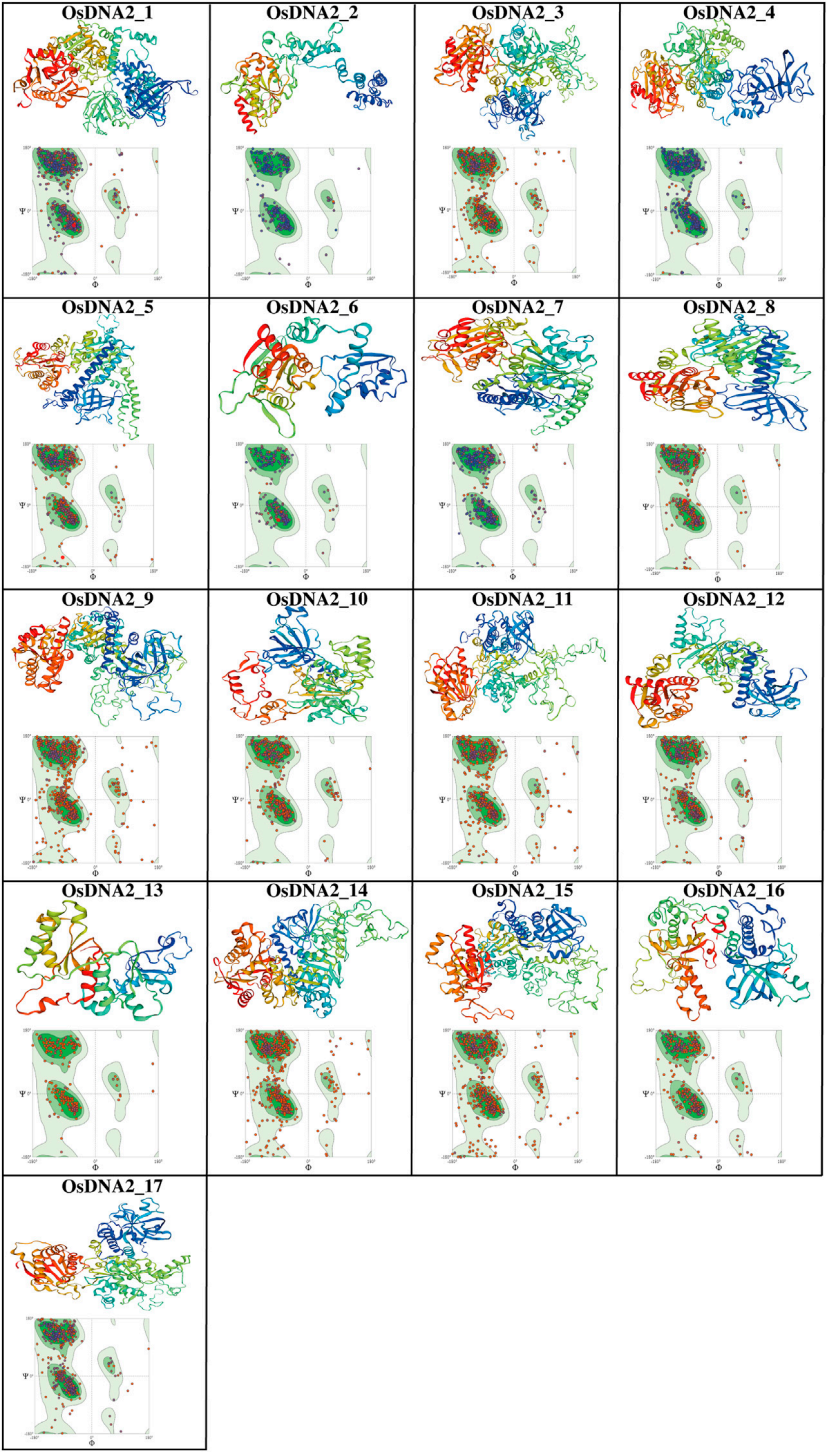
The 3D structure prediction and Ramachandran plots of OsDNA2 proteins. Different shapes in these final models represent the sheets and helicases.

Understanding the structure of proteins is very difficult due to their complexity, and the fact that they contain a different number of atoms and convoluted topology. In this study, the SWISS_MODEL online server was used to predict the three-dimensional (3D) structures of 17 OsDNA2 proteins (Figure 8, Supplementary Table S7). The results revealed the successful prediction of the 17 OsDNA2 protein models. Many reports have shown that >30% identity with the template is acceptable (Xiang, 2006; Rahim et al., 2022), and our findings also showed an average of 40% similarity with the template (5ean. 1.A, 6ff7. 1.c, 5mzn. 1.A, 2wjv. 1.A, 4b3f. 1.A, 4b3g. 2.A, 2gjk. 1.A, 2wjv. 1.A, and 2xzl. 1.A). Spiral shapes represent α-helices, thick arrows depict ß-sheets, and thin lines indicate loops and turns. Most of the OsDNA2 members had similar structures (Figure 8). Furthermore, these predicted 3D models were confirmed using Ramachandran plots with the help of diahedral angles of the OsDNA2 proteins (Anderson et al., 2005). The results of the Ramachandran plots revealed that >95% of the regions of OsDNA2 proteins showed highly favorable regions. This confirmed the better quality and stability of the predicted OsDNA2 protein structures.

### Role of *OsDNA2* genes in abiotic stress tolerance

**FIGURE 9:**
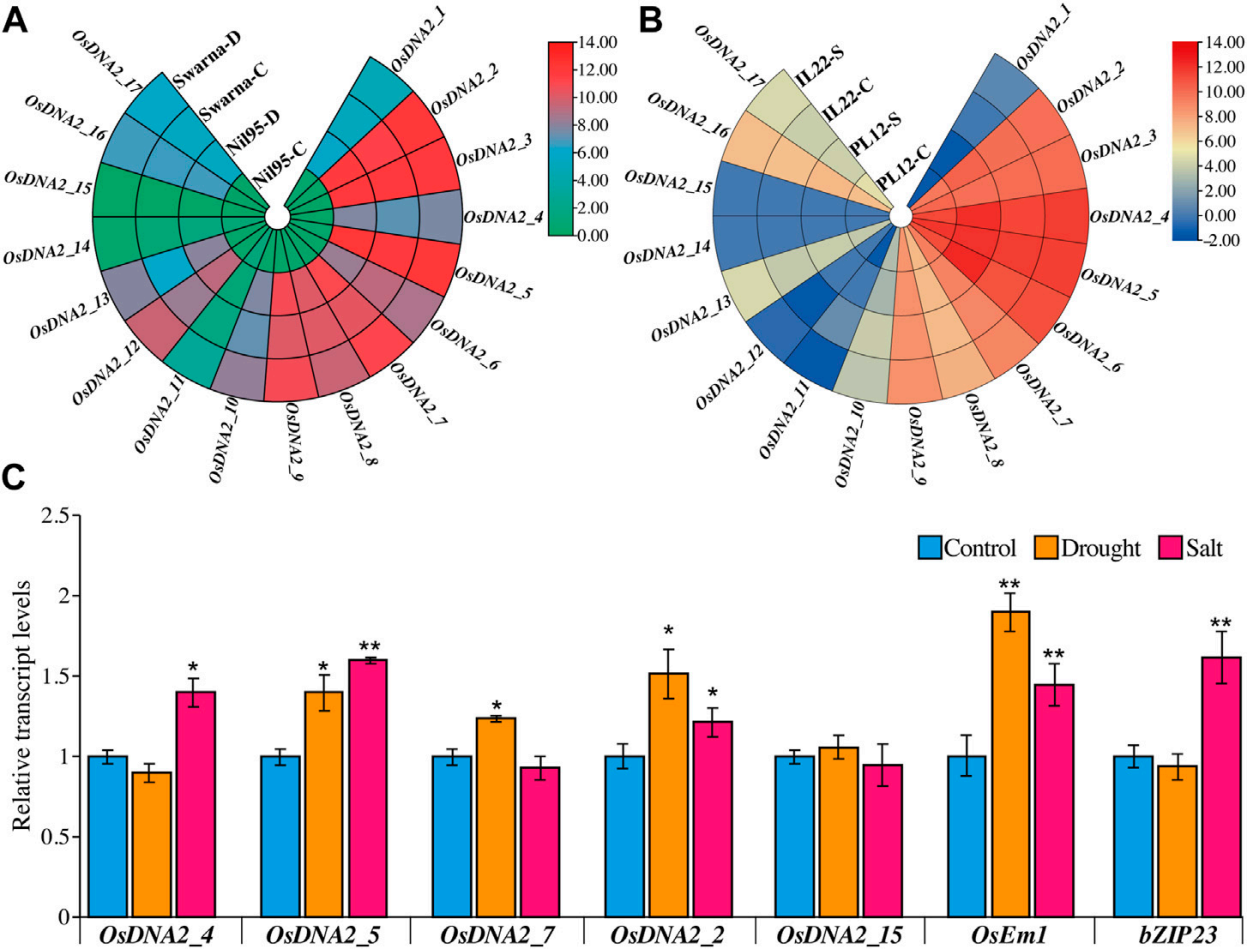
9In-silico expression profiling of OsDNA2 genes members under drought **(A)** and salt **(B)** stress. Different capital letters C, D, and S represent the control, drought, and salt stresses, respectively. **(C)** Quantification of expression of OsDNA2 genes through qRT-PCR. Results are shown as fold change. Data is a mean ± SE of three biological replicates. Comparison was checked with the help of t-test (*p < 0.05, **p < 0.01).

RNA-seq data for drought and salt stress were retrieved from publicly available data sites. Circular heat maps for these stresses were generated from the FPKM values, showing the expression of *OsDNA2* members at the seedling stage (Figures 9A,B, Supplementary Table S8). *OsDNA2_11*, *OsDNA2_14,* and *OsDNA2_15* were downregulated and showed low expression under drought and salt stress conditions (Figures 9A,B). Quantitative real-time PCR (qRT-PCR) analysis of five randomly selected *OsDNA2* genes was performed to study the transcription profile (Figure 9C). *OsDNA2_2* and *OsDNA2_5* were up-regulated under both stress conditions*,* whereas *OsDNA2_4* and *OsDNA2_7* were up-regulated under salt and drought stress, respectively. Moreover, *OsDNA2_15* expression was unchanged after the treatments. These findings indicate that these genes might be involved in mitigating abiotic stresses (drought and salt), and they may provide useful information for the functional characterization of these genes in the future.

## Discussion

The DNA of a living organism contains genetic information that is important for its survival and reproduction. During cell division, DNA strands can be damaged by abiotic stresses such as exposure to radiation or chemicals. To avoid the consequent negative impacts on life, cells initiate processes that quickly fix damaged DNA using different techniques, such as homologous recombination, in which cells fix the damaged strands (Duxin et al., 2009). Different enzymes act on broken strands to form single-stranded tails, and DNA2 is one of the enzymes involved (Jia et al., 2016). In yeast, the *dna2* mutant shows a lethal phenotype, indicating that the wild-type protein is necessary for cell viability (Budd et al., 2011). Similarly, the Arabidopsis *dna2* mutant caused small roots at the time of germination (Diray-Arce et al., 2013; Jia et al., 2016). Another protein, Rpa, is required for the proper functioning of DNA2; however, it remains unclear how Rpa regulates it. DNA2 homologs are the same in fungi and other metazoans, indicating that they may have conserved functions (Kang et al., 2010). In plants, the alternate DNA helicase is DNA2 (Levikova and Cejka, 2015). Plants face different types of biotic and abiotic stresses that cause cell injury, DNA damage including double- and single-stranded breaks (DSBs and SSBs), and DNA lesions (Gill et al., 2015; Noctor and Foyer, 2016; Nisa et al., 2019; Rout et al., 2022). Because the function of DNA2 is conserved in DNA damage repair and Okazaki fragments, the small RNA/DNA was removed by *FEN1*, while the larger flaps were removed in the correct order of both FEN1 and DNA2 (Kang et al., 2010; Zheng and Shen, 2011). To date, DNA2 has mainly been studied in fungi and animals, but its role in plants has not been fully studied. Therefore, this study aimed to investigate the function of DNA2 in rice at the whole-genome level.

Rice is an important cereal used worldwide as a good source of food around the globe. Rice production is hampered by biotic and abiotic stresses. The rice genome is publicly available, which permits genome-wide identification and characterization of the DNA2 family (Table 1). In this study, we identified 17 *OsDNA2* members in the rice genome (Figure 1A) that were distributed into three clusters. The expression of any gene is dependent on its structure. In this study, gene structure analysis confirm that 2 to 33 exons were present among the *OsDNA2* genes, whereas the number of introns varied from 1 to 32 (Figure 1B). Conserved motifs and domain analyses were also carried out systematically. The AAA_11 superfamily (Pfam: PF13086) domain is present in the *OsDNA2* gene, which belongs to the DEAD-like helicase superfamily, and is involved in ATP-dependent RNA or DNA unwinding (Figure 2). Phylogenetic association was also carried out among different plant species, such as *Oryza sativa*, *Hordeum vulgare*, *Zea mays*, *Sorghum bicolor*, and *Arabidopsis thaliana* (Figure 3). These results indicate that OsDNA2s were conserved with related species, and this may be due to the presence of conserved domains among them. Similarly, we performed synteny and collinearity analyses among *Oryza sativa*, *Sorghum bicolor*, *Zea mays*, and *Hordeum vulgare*. The results revealed that rice *DNA2* genes were more collinear with sorghum than with maize and barley, indicating that segmental duplication is involved in *OsDNA2* gene family progression (Figure 4B).

To better understand the function of *OsDNA2* genes in stress tolerance, *cis*-regulatory elements were predicted (Figure 5). Our results showed that three main types of *cis*-elements (stress, hormones, and light) were detected. Among these stress-related regulatory elements, CGTCA-motif, G-box, Sp1, GATA-motif, I-box, GT1-motif, and AT-rich were detected. According to previous studies, *cis*-elements are involved in the stress response (Rasool et al., 2021; Su et al., 2021). The GCN4-motif, GC-motif, O2-box, and ARE were detected in the different *OsDNA2* members, indicating that these genes are involved in cellular development. These results were further confirmed through gene ontology (Figure 6), in which most of the GO terms were related to DNA replication, DNA helicase, and catalytic and ATP-dependent activities. These findings further emphasized the thought that *OsDNA2* genes may be involved in DNA repair and replication under environmental stress.

Recent studies have shown that most plant biological processes are controlled by microRNAs through the regulation of gene expression (Millar, 2020). In grasses, different miRNAs are expressed under drought conditions (Njaci et al., 2018; Raza et al., 2022). In another study on Arabidopsis, miRNA394 was shown to respond to cold stress. Similarly, in wheat, different miRNAs, such as tae-miR1119, tae-miR398, and tae-miR444a, were expressed in the roots under drought conditions (Rasool et al., 2021). In this study, we identified 653 miRNAs that target 17 *OsDNA2* genes (Figure 7). To date, the highest number of identified miRNAs is 1077 in maize (Fu et al., 2017). These miRNAs can be upregulated or downregulated in response to environmental stress. In these findings, *OsDNA2_4* was targeted by 74 miRNAs, and was upregulated under salt stress.

For further in-depth study, we predicted 3D models of the OsDNA2 proteins (Figure 8). The results showed 20–74% homology with the templates, which is widely acceptable. Our findings are in accordance with those of other studies (Su et al., 2021; Rahim et al., 2022). The Ramachandran plots also confirmed that the 17 OsDNA2 proteins had favorable regions because they showed >80% residue in the allowed regions, which indicates that the predicted 3D structures of these proteins were of good quality. Similar findings have also been reported for TATrx and TaRPK1 proteins in wheat (Bhurta et al., 2022; Rahim et al., 2022). In another study, 21 TaEIL 3D models and Ramachandran plots were used (Yi-Qin et al., 2020).

Previously, it was reported in mammals and other microorganisms that *DNA2* genes are expressed differently under different circumstances. Thus, in this study, we examined the expression of *OsDNA2* genes in plants under control, drought, and salt stress conditions. We found that some of the *OsDNA2* genes were up- and downregulated under drought and salt stress conditions (Figures 9A,B). qRT-PCR of five randomly selected *OsDNA2* genes (*OsDNA2_2, OsDNA2_4, OsDNA2_5, OsDNA2_7,* and *OsDNA2_15*), along with well-known drought- (*OsEm1*) and salt- (*bZIP23*) related genes, was performed to study the transcription profile (Figure 9C). *OsDNA2_2* and *OsDNA2_5* were upregulated under both stress conditions*,* whereas *OsDNA2_4* and *OsDNA2_7* were up-regulated under salt and drought stress, respectively. However, *OsDNA2_15* expression was unchanged after treatment. These findings suggest that these genes are involved in abiotic stress mitigation. Moreover, this study also signifies the need for the functional characterization of these genes in the near future.

## Conclusion

In animals and yeast *DNA2* are well characterized because it has the abilities of both helicase and nuclease, it plays a crucial role in DNA replication in the nucleus and mitochondrial genomes. However; they are not fully examined in plants due to less focused on plants damage repair. The current study extensively examined the characteristic, properties, gene structures, chromosomal locations, *cis*-regulatory elements, synteny and collinearity, miRNAs, and expression of *OsDNA2*. Overall, 17 *OsDNA2* genes were reported in the whole genome of rice, and were distributed on eight chromosomes. Phylogenetic analysis revealed that all the *OsDNA2* genes were organized into three groups. We also found that the conserved domain (AAA_11 superfamily) was present in the *OsDNA2* genes, which belongs to the DEAD-like helicase superfamily. In addition, 653 miRNAs targeting *OsDNA2* genes were identified. Meanwhile, shifts in gene expression under abiotic stress, especially drought and salinity, were investigated using a comparative transcriptome approach to evaluate the susceptibility and tolerance to abiotic stress. These findings provide essential information for future functional characterization of *OsDNA2* genes under abiotic stress to improve stress tolerance in rice as well as other crop species.

## Data Availability

The original contributions presented in the study are included in the article/Supplementary Material, further inquiries can be directed to the corresponding authors.
