# Supplementary material for: Genome-wide and molecular characterization of the DNA replication helicase 2 (DNA2) gene family in rice under drought and salt stress
Source: Front Genet. 2022 Nov 22;13:1039548. doi: 10.3389/fgene.2022.1039548 (PMC9728955; doi:10.3389/fgene.2022.1039548)
Supplement: Supplementary file 1 [file DataSheet1.ZIP › Supplimentary data/Supplementary Table S3. Gene duplication Ka:Ks.docx]

**Supplementary Table S3:** Gene duplication events.

| **Gene-1** | **Gene-2** | **Ka** | **Ks** | **Ka/Ks** | **Type of Duplication** |
| --- | --- | --- | --- | --- | --- |
| *OsDNA2_5* | *OsDNA2_6* | 0.2317 | 0.2836 | 0.8169 | Segmental |
| *OsDNA2_16* | *OsDNA2_17* | 0.1396 | 0.2766 | 0.5046 | Segmental |
| *OsDNA2_1* | *OsDNA2_8* | 0.8347 | 0.5313 | 1.5710 | Segmental |
| *OsDNA2_14* | *OsDNA2_2* | 0.9043 | 0.4728 | 1.9126 | Segmental |
| *OsDNA2_12* | *OsDNA2_13* | 0.4158 | 0.4528 | 0.9182 | Segmental |
| *OsDNA2_10* | *OsDNA2_11* | 0.4834 | 0.3241 | 1.4915 | Segmental |
