# Supplementary material for: Genome-wide and molecular characterization of the DNA replication helicase 2 (DNA2) gene family in rice under drought and salt stress
Source: Front Genet. 2022 Nov 22;13:1039548. doi: 10.3389/fgene.2022.1039548 (PMC9728955; doi:10.3389/fgene.2022.1039548)
Supplement: Supplementary file 1 [file DataSheet1.ZIP › Supplimentary data/Supplementary Table S1. Primers..docx]

Supplementary Table S1. List of primers used for quantitative real-time PCR.

| **Primer name** |  | **Primer sequence (5´-3´)** |
| --- | --- | --- |
| *Os07g0495900* | F | AGCCAGAGCAAAGATAGCC |
|  | R | ACAAAATCCACGCTAAAGCC |
| *Os09g0130800* | F | CTTCCATTCCCATCTTTTCCC |
|  | R | TCTCCTTCCTCTTTCTCCCC |
| *Os03g0586900* | F | TCTTGCTTGTGCTGCTTC |
|  | R | GCTGTTGTCGTTTTCTCTCTTC |
| *Os03g0387000* | F | ACTCCACAACCTACCTTCAC |
|  | R | CTCAAAAGCCCTACATTCTTCC |
| *Os02g0684150* | F | GGGAAGACGAAGACCATCAG |
|  | R | CCGAACAGCACAACATCAC |
| *OsEm1* | F | GAGGAAGGGTACCGCGAGAT |
|  | R | GTCTTGTACTTGGACTCGTCG |
| *OsbZIP23* | F | GGAGCAGCAAAAGAATGAGG |
|  | R | GGTCTTCAGCTTCACCATCC |
| *OsActin* | F | TCCATCTTGGCATCTCTCAG |
|  | R | GGTACCCTCATCAGGCATCT |
