# Supplementary figures and images for: Genome-wide and molecular characterization of the DNA replication helicase 2 (DNA2) gene family in rice under drought and salt stress
Source: Front Genet. 2022 Nov 22;13:1039548. doi: 10.3389/fgene.2022.1039548 (PMC9728955; doi:10.3389/fgene.2022.1039548)

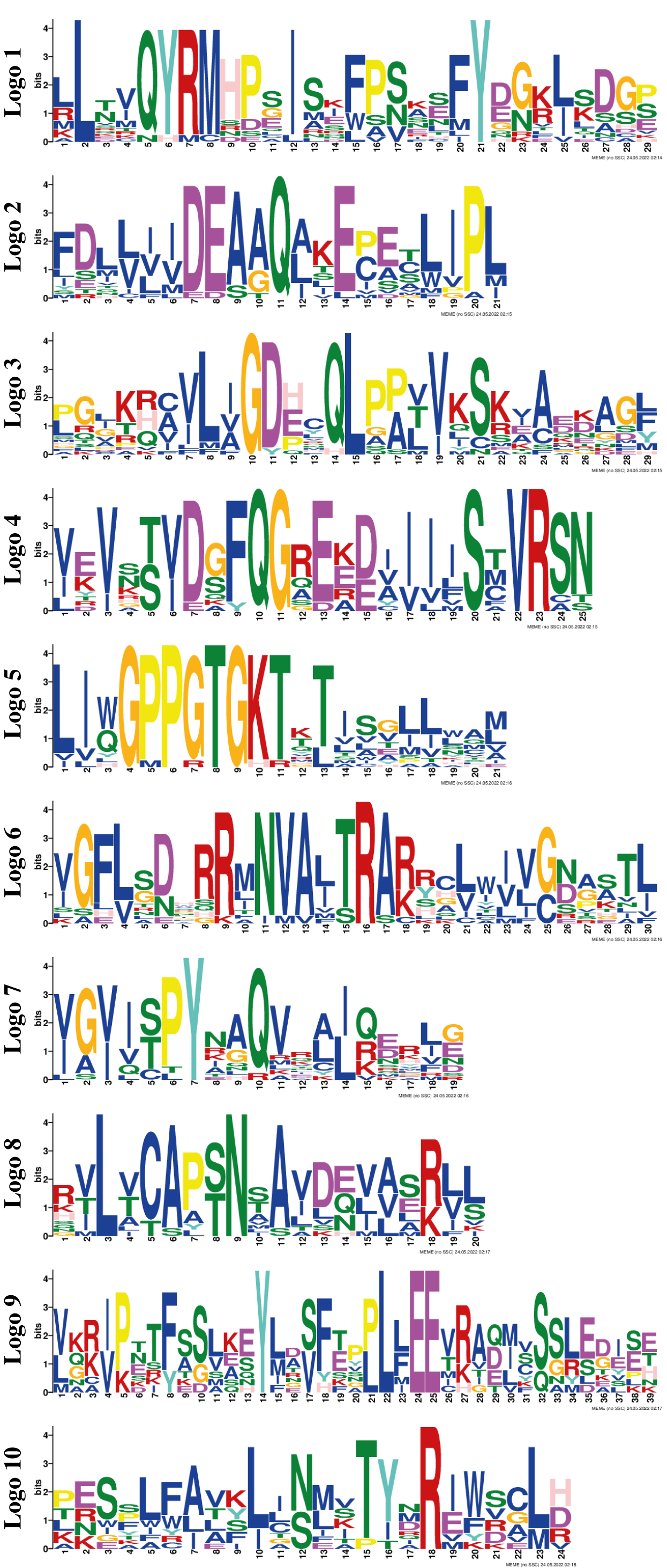

Supplement: Supplementary file 1 [file DataSheet1.ZIP › Supplimentary data/Supplementary Figure S1. Logos.tif]
